# Association between the severity of interstitial lung disease and the risk of osteoporotic vertebral fractures in patients with rheumatoid arthritis

**DOI:** 10.1097/MD.0000000000048032

**Published:** 2026-03-27

**Authors:** Na Li, Jiaxin Li

**Affiliations:** a Department of Rheumatology and Immunology, Xuzhou Central Hospital, Xuzhou City, Jiangsu Province, China.

**Keywords:** bone mineral density, interstitial lung disease, multivariable regression, osteoporotic vertebral fracture, rheumatoid arthritis

## Abstract

This study aimed to investigate the relationship between interstitial lung disease (ILD) severity and bone mineral density (BMD) and osteoporotic vertebral fractures (OVFs) in patients with rheumatoid arthritis (RA) and determine whether ILD independently affects skeletal health. This retrospective cross-sectional study included 150 RA patients treated between January 2021 and December 2024. Based on high-resolution chest computed tomography, patients were classified as RA-ILD (n = 62) or RA without ILD (n = 88). RA-ILD cases were further stratified by fibrosis extent into mild (<20%, n = 27), moderate (20%–50%, n = 24), and severe (>50%, n = 11). Demographic data, clinical features, inflammatory markers, vitamin D, bone turnover indices, and medication exposure, including cumulative glucocorticoid dose, were collected. BMD at the lumbar spine, total hip, and femoral neck was measured by dual-energy X-ray absorptiometry. OVF was assessed by thoracolumbar radiography or computed tomography using the Genant semiquantitative method. Group comparisons, trend analyses, correlation analyses, and multivariable logistic regression were performed to identify independent risk factors for OVF. ILD was present in 41.3% of RA patients. Compared with those without ILD, RA-ILD patients were older, had longer disease duration, higher disease activity, elevated C-reactive protein/erythrocyte sedimentation rate, lower 25-hydroxyvitamin D, higher bone alkaline phosphatase levels, and greater cumulative glucocorticoid exposure (all *P* < .05). Lumbar spine, total hip, and femoral neck BMD were significantly lower in the RA-ILD group, accompanied by a lower lumbar T-score. OVF prevalence was higher in RA-ILD patients (30.6% vs 14.8%), including more multiple fractures. Within the RA-ILD cohort, BMD declined progressively with increasing ILD severity, and pulmonary function indices (forced vital capacity [%] and diffusing capacity for carbon monoxide [%]) showed positive correlations with BMD. OVF frequency rose stepwise from mild to severe ILD (14.8%, 29.2%, and 54.5%). Multivariable analysis identified ILD severity as an independent predictor of OVF (odds ratios = 2.51, 95% confidence interval: 1.21–5.22), together with age and cumulative glucocorticoid exposure. RA patients with ILD exhibit significantly reduced BMD and an increased risk of OVFs, which worsen with ILD severity. ILD appears to be an independent risk factor for skeletal deterioration in RA, highlighting the importance of early bone health assessment and preventive management in this population.

## 1. Introduction

Rheumatoid arthritis (RA) is a systemic autoimmune disease characterized by chronic synovial inflammation and immune dysregulation, with a high risk of osteoporosis (OP) and fractures.^[1,2]^ Previous studies have shown that RA patients have significantly lower bone mineral density (BMD) than the general population, and the incidence of vertebral fractures is increased by 1.5- to 2-fold. This risk is closely associated not only with chronic inflammation and glucocorticoid exposure but also with cytokine-mediated disruptions in bone metabolism.^[3,4]^ In addition, extra-articular manifestations are common in RA, among which interstitial lung disease (ILD) is the most prevalent, affecting approximately 20% to 30% of patients, and it has emerged as a major contributor to RA-related mortality.^[5,6]^

Recent evidence suggests that pulmonary fibrosis and bone metabolic abnormalities may share common pathological pathways, including inflammatory cytokines (TNF-α and IL-6), oxidative stress, and activation of TGF-β signaling, all of which may accelerate bone resorption and inhibit bone formation.^[7–9]^ Furthermore, RA-ILD patients often experience reduced physical activity, impaired nutritional status, and long-term glucocorticoid use, which may exacerbate bone loss. However, existing studies on the relationship between RA-ILD and the osteoporotic vertebral fracture (OVF) risk remain limited. Most investigations focus on BMD differences, and few have explored the dose-response relationship between ILD severity and skeletal health.

ILD exhibits considerable heterogeneity among RA patients, with variations in disease duration and severity potentially affecting systemic inflammation, pulmonary gas exchange, and physical capacity, thereby influencing bone microarchitecture and fracture risk.^[10,11]^ Therefore, clarifying the association between ILD severity and both BMD and OVF risk in RA patients is critical for improving clinical risk stratification and guiding precision management.

Based on these considerations, this study retrospectively analyzed clinical and imaging data of RA patients, to compare BMD and OVF incidence between RA patients with and without ILD, explore the relationship between ILD severity and BMD as well as OVF risk, and construct multivariable regression models to evaluate the independent effect of ILD on OVF. The findings aim to provide evidence-based guidance for bone health management in RA-ILD patients.

## 2. Materials and methods

### 2.1. Study population

This study was approved by the Ethics Committee of Xuzhou Central Hospital. This single-center retrospective cohort study included 150 patients with confirmed RA who were admitted to the Department of Rheumatology and Immunology between January 2021 and December 2024 and completed all relevant examinations. Clinical data, imaging results, laboratory parameters, and follow-up information were obtained from the hospital electronic medical record system and picture archiving and communication system imaging database. The study protocol was approved by the institutional ethics committee and conducted in accordance with the Declaration of Helsinki. All patients provided written informed consent at admission, agreeing to the use of anonymized clinical data for research purposes. To ensure privacy, all data were anonymized prior to analysis and contained no personally identifiable information. Based on high-resolution chest computed tomography (HRCT) findings, patients were categorized into RA-ILD (n = 62, 41.3%) and RA-non-ILD (n = 88, 58.7%) groups.

### 2.2. Inclusion and exclusion criteria

The inclusion criteria were as follows: age ≥ 18 years; fulfillment of the 2010 American College of Rheumatology/European Alliance of Associations for Rheumatology RA classification criteria; at least 1 HRCT scan to evaluate the presence and extent of ILD; completion of dual-energy X-ray absorptiometry (DXA) for BMD during the study period; availability of complete clinical and laboratory data; and provision of informed consent for the use of medical data for research.

The exclusion criteria were as follows: comorbidities affecting bone metabolism, such as hyper/hypothyroidism, hyperparathyroidism, or Cushing syndrome; malignancy, advanced chronic kidney disease (stage ≥ 4), or severe chronic liver disease; history of traumatic or high-energy fractures within the past year; prior use of anti-osteoporotic medications for ≥12 months (e.g., bisphosphonates, denosumab, and teriparatide); co-existing conditions known to cause ILD, such as systemic sclerosis, idiopathic pulmonary fibrosis, or occupational lung disease; and incomplete clinical or follow-up data.

### 2.3. Assessment of ILD severity

#### 2.3.1. Imaging acquisition protocol

All participants underwent HRCT for ILD assessment. HRCT was performed in the supine position with full inspiration (and end-expiratory phase if indicated), covering the lung apices to the diaphragmatic domes. Slice thickness was ≤1.5 mm (recommended 1.0 mm) with matching intervals, reconstructed using a high-frequency algorithm (bone/edge-enhancing kernel) to optimize lung parenchyma visualization. Scan parameters included tube voltage 120 kVp, automatic or body-size-adjusted tube current, and manufacturer-recommended pitch and rotation time. Images were archived in the hospital picture archiving and communication system and reviewed using standard lung (width 1500 HU, level −600 HU) and mediastinal windows. To maintain temporal relevance, HRCT and DXA were performed within ±3 months.

#### 2.3.2. Semiquantitative scoring: Warrick method

ILD severity was assessed using the semi-quantitative Warrick scoring system, a validated method widely applied in clinical studies. The score incorporates both the type and extent of HRCT abnormalities, including ground-glass opacities, reticular changes/traction bronchiectasis, and honeycombing/lower-lobe fibrosis. Each lesion type and distribution is assigned predefined points, and the cumulative involvement of lung segments/lobes is quantified to calculate a total score ranging from 0 to 30. For analysis, patients were stratified by total Warrick score into mild (≤7), moderate (8–15), and severe (≥16) ILD groups for subsequent comparisons and statistical analyses.

### 2.4. BMD and fracture assessment

#### 2.4.1. BMD measurement and quality control

BMD of the lumbar spine and hip was assessed using DXA. All measurements were performed on the same DXA device (Hologic Discovery A or equivalent) by certified technologists with ≥3 years of experience. Scanned sites included the lumbar spine (L1–L4, anteroposterior projection), femoral neck, and total hip. BMD (g/cm^2^) and T-scores were recorded. According to the World Health Organization criteria, bone status was classified as: normal (*T* ≥ −1.0), osteopenia (−2.5 < *T* < −1.0), or OP (*T* ≤ −2.5). To ensure measurement consistency, quality control procedures included device calibration at study initiation, daily automated self-checks, and periodic phantom scanning. DXA technologists underwent protocol-specific training to standardize operations. In cases where metallic implants or spinal deformities interfered with lumbar BMD measurement, contralateral hip or peripheral sites (e.g., forearm) were used as substitutes, with annotations in the dataset. The temporal interval between BMD assessment and HRCT was limited to ±3 months.

#### 2.4.2. Fracture definition and imaging diagnosis

OVFs were assessed using standard anteroposterior and lateral thoracolumbar radiographs, supplemented with HRCT 3D reconstruction if necessary. Fractures were graded using the Genant semiquantitative method: mild (20%–25% vertebral height loss), moderate (25%–40%), and severe (>40%). Vertebral morphology was independently evaluated by 2 blinded radiologists or orthopedic physicians, each with ≥5 years of experience. Discrepancies exceeding 1 grade or disagreement on fracture presence were adjudicated by a senior expert to reach a consensus. Exclusion criteria included traumatic fractures, infectious or neoplastic vertebral destruction, and congenital spinal deformities. For patients with prior vertebral fractures, imaging was reviewed to distinguish new fractures occurring within the study window; if uncertain, they were recorded as historical fractures and excluded from incident fracture analysis.

### 2.5. Clinical and laboratory data

#### 2.5.1. Baseline clinical information

Baseline clinical data were extracted from the electronic medical record system and patient interviews at enrollment. Collected variables included demographic characteristics (age, sex, body mass index [BMI], smoking and alcohol history), disease features (RA duration, joint involvement, morning stiffness, and tender/swollen joint counts), functional status and disease activity (Health Assessment Questionnaire-Disability Index, Disease Activity Score in 28 joints using CRP [DAS28-CRP]), and fall history and muscle strength (falls within the previous 12 months, handgrip strength if applicable). Data collection followed standardized case report forms and were independently verified by 2 trained researchers to ensure accuracy.

#### 2.5.2. Laboratory measurements

Fasting venous blood samples were collected within 24 hours of enrollment. Laboratory assessments included inflammatory and immune markers: C-reactive protein (CRP), erythrocyte sedimentation rate (ESR), immunoglobulins (IgG, IgA, IgM), rheumatoid factor (RF), anti-cyclic citrullinated peptide (anti-CCP) antibodies; bone metabolism-related markers: 25-hydroxyvitamin D, serum calcium, phosphorus, bone-specific alkaline phosphatase; and routine biochemical markers: complete blood count, liver and renal function, electrolytes, fasting glucose, and lipid profile. All laboratory tests were performed in the hospital clinical laboratory under ISO 15189 quality standards, with periodic equipment calibration and internal quality control.

#### 2.5.3. Medication and treatment data

Treatment information was systematically recorded, including glucocorticoid use (daily dose, cumulative dose, and duration), conventional synthetic disease-modifying antirheumatic drugs (methotrexate, leflunomide, hydroxychloroquine, etc), biologic and targeted synthetic disease-modifying antirheumatic drugs (TNF inhibitors, IL-6 inhibitors, JAK inhibitors, etc), nonsteroidal anti-inflammatory drugs, and bone-protective therapies (calcium, vitamin D, bisphosphonates, strontium ranelate, denosumab, and teriparatide). Cumulative glucocorticoid doses were automatically extracted from the medical and prescription records and manually verified for accuracy.

### 2.6. Statistical analysis

All statistical analyses were performed using SPSS software version 26.0 (IBM Corp., Armonk), with a two-sided significance threshold of *P* < .05. Continuous variables were first tested for normality. Normally distributed data are presented as mean ± standard deviation and compared using independent-samples *t* tests or one-way analysis of variance as appropriate. Non-normally distributed data are expressed as median with interquartile range and compared using the Mann–Whitney *U* test or Kruskal–Wallis *H* test. Categorical variables are reported as frequencies and percentages, with comparisons performed using the χ^2^ test or Fisher exact test as appropriate. ILD severity (mild, moderate, and severe) was treated as a categorical independent variable, and BMD (T-scores) and OVF incidence were defined as primary outcome measures. To evaluate the independent association between ILD severity and OVF risk in RA patients, multivariable logistic regression models were constructed. Covariates potentially influencing bone metabolism and fracture risk – including age, sex, RA disease duration, BMI, smoking history, cumulative glucocorticoid dose, RF and anti-CCP antibody status, inflammatory markers (CRP, ESR), and use of bone-protective therapy – were included. Stepwise regression was applied to identify significant predictors, with adjusted odds ratios (ORs) and 95% confidence intervals (CIs) reported. Linear regression analyses were performed to examine the relationships between ILD severity and continuous BMD measures.

## 3. Results

### 3.1. Baseline clinical characteristics

A total of 150 patients with RA were included in this study, of whom 62 (41.3%) were classified as RA with ILD (RA-ILD) and 88 (58.7%) as RA without ILD (RA-non-ILD). No significant differences were observed between the 2 groups in terms of sex distribution, BMI, smoking, or alcohol consumption (all *P* > .05). The overall cohort was predominantly female (72.7%) with a mean age of 62.6 ± 9.5 years. Compared with the RA-non-ILD group, RA-ILD patients were older (64.9 ± 8.7 vs 61.2 ± 9.9 years, *P* = .011) and had a longer disease duration (8.0 [5.0–12.0] vs 6.0 [4.0–10.0] years, *P* = .030). Disease activity and functional impairment were more pronounced in the RA-ILD group, as evidenced by higher DAS28-CRP and Health Assessment Questionnaire-Disability Index scores (both *P* < .05). Although the proportion of patients with a history of falls in the preceding 12 months was slightly higher in the ILD group, the difference was not statistically significant (*P* = .129). Regarding inflammatory and immune parameters, RA-ILD patients exhibited significantly elevated CRP (11.5 [6.3–22.0] mg/L vs 7.9 (4.1–16.5) mg/L, *P* = .019) and ESR levels (42.2 ± 18.6 vs 36.0 ± 17.8 mm/h, *P* = .044) compared with the RA-non-ILD group. The prevalence of RF and anti-CCP antibody positivity was higher in the RA-ILD group, although the differences did not reach statistical significance (both *P* > .05). In terms of bone metabolism, RA-ILD patients had lower 25-hydroxyvitamin D levels and higher bone-specific alkaline phosphatase levels (both *P* < .05), indicating more pronounced bone metabolic imbalance. Cumulative glucocorticoid exposure was significantly higher in the RA-ILD group (5.1 [3.2–7.8] g vs 3.8 [2.5–6.0] g, *P* = .008). There were no significant differences in the use of conventional synthetic disease-modifying antirheumatic drugs, biologic disease-modifying antirheumatic drugs, or targeted synthetic disease-modifying antirheumatic drugs between groups (all *P* > .05). The proportion of patients receiving anti-osteoporotic therapy was slightly higher in the RA-ILD group (32.3% vs 27.3%, *P* = .468). Overall, RA-ILD patients were characterized by older age, higher inflammatory burden, longer disease duration, greater functional impairment, and higher cumulative glucocorticoid exposure, providing a robust basis for subsequent analyses of the association between ILD severity and vertebral fracture risk (Table 1).

**Table 1:** Baseline characteristics of RA patients with and without ILD.

Characteristics	Total (n = 150)	RA-ILD (n = 62)	RA-non-ILD (n = 88)	*P* value
Demographic characteristics				
Age, yr	62.6 ± 9.5	64.9 ± 8.7	61.2 ± 9.9	.011*
Female, n (%)	109 (72.7)	46 (74.2)	63 (71.6)	.732
BMI, kg/m^2^	22.7 ± 3.3	22.4 ± 3.1	22.9 ± 3.4	.342
Smoking history, n (%)	41 (27.3)	19 (30.6)	22 (25.0)	.446
Drinking history, n (%)	33 (22.0)	14 (22.6)	19 (21.6)	.882
Disease characteristics				
Disease duration, yr	7.0 (4.0–11.0)	8.0 (5.0–12.0)	6.0 (4.0–10.0)	.030*
Morning stiffness, min	45 (30–70)	50 (35–75)	40 (25–60)	.041*
Tender joint count	6 (4–9)	7 (5–10)	5 (3–8)	.036*
Swollen joint count	4 (2–7)	5 (3–8)	3 (2–6)	.048*
DAS28-CRP	4.21 ± 1.09	4.52 ± 1.15	3.99 ± 0.98	.004*
HAQ-DI	1.02 ± 0.56	1.18 ± 0.61	0.90 ± 0.50	.008*
Fall history in last 12 mo, n (%)	31 (20.7)	16 (25.8)	15 (17.0)	.129
Grip strength, kg	20.8 ± 6.5	19.7 ± 6.3	21.5 ± 6.6	.118
Laboratory indicators				
CRP, mg/L	9.3 (4.8–18.6)	11.5 (6.3–22.0)	7.9 (4.1–16.5)	.019*
ESR, mm/h	38.8 ± 18.3	42.2 ± 18.6	36.0 ± 17.8	.044*
RF positive, n (%)	121 (80.7)	53 (85.5)	69 (78.4)	.266
Anti-CCP positive, n (%)	118 (78.7)	52 (83.9)	68 (77.3)	.318
25(OH)D, ng/mL	18.7 ± 6.2	17.2 ± 5.8	19.8 ± 6.4	.020*
BALP, U/L	18.9 ± 6.8	20.6 ± 7.1	17.8 ± 6.5	.032*
Treatment characteristics				
Cumulative glucocorticoid dose, g	4.3 (2.8–6.9)	5.1 (3.2–7.8)	3.8 (2.5–6.0)	.008*
csDMARDs use, n (%)	135 (90.0)	56 (90.3)	79 (89.8)	.927
b/tsDMARDs use, n (%)	42 (28.0)	18 (29.0)	24 (27.3)	.814
Anti-osteoporosis therapy, n (%)	43 (28.7)	20 (32.3)	23 (27.3)	.468

*P* < 0.05 indicates statistical significance.

25(OH)D = 25-hydroxyvitamin D, BALP = bone-specific alkaline phosphatase, bDMARDs = biologic DMARDs, BMI = body mass index, CCP = cyclic citrullinated peptide, CRP = C-reactive protein, csDMARDs = conventional synthetic disease-modifying antirheumatic drugs, DAS28-CRP = Disease Activity Score 28-CRP, ESR = erythrocyte sedimentation rate, HAQ-DI = Health Assessment Questionnaire-Disability Index, ILD = interstitial lung disease, IQR = interquartile range, RA = rheumatoid arthritis, RF = rheumatoid factor, SD = standard deviation, tsDMARDs = targeted synthetic DMARDs.

* Data are presented as mean ± SD, median (IQR), or n (%).

### 3.2. BMD and OVF incidence

Compared with RA patients without ILD, those with RA-ILD exhibited more pronounced bone loss at multiple skeletal sites. DXA measurements revealed significantly lower BMD in the RA-ILD group at the lumbar spine (L1–L4; 0.852 ± 0.114 vs 0.908 ± 0.121 g/cm^2^, *P* = .006), total hip (0.784 ± 0.102 vs 0.826 ± 0.098 g/cm^2^, *P* = .017), and femoral neck (0.679 ± 0.089 vs 0.713 ± 0.092 g/cm^2^, *P* = .028). Correspondingly, T-scores were also lower in the RA-ILD group, indicating a heavier osteoporotic burden. Specifically, lumbar spine T-scores were −2.19 ± 0.78 in RA-ILD patients versus −1.86 ± 0.72 in RA-non-ILD patients (*P* = .009), and total hip T-scores were −1.84 ± 0.69 versus −1.55 ± 0.63 (*P* = .014). Regarding vertebral fractures, the incidence of OVFs was significantly higher in the RA-ILD group than in the RA-non-ILD group (30.6% [19/62] vs 14.8% [13/88], *P* = .018). Further stratification by the number of fractures demonstrated that the proportion of patients with multiple vertebral fractures was greater in the RA-ILD group (12.9% vs 4.5%, *P* = .045), suggesting that RA-ILD patients not only have lower BMD but also a higher fracture burden (Table 2).

**Table 2:** Comparison of bone mineral density and osteoporotic vertebral fractures between RA-ILD and RA-non-ILD groups.

Parameter	RA-ILD (n = 62)	RA-non-ILD (n = 88)	Test statistic	*P* value
Lumbar spine BMD (g/cm^2^)	0.852 ± 0.114	0.908 ± 0.121	*t* = 2.79	.006*
Total hip BMD (g/cm^2^)	0.784 ± 0.102	0.826 ± 0.098	*t* = 2.41	.017*
Femoral neck BMD (g/cm^2^)	0.679 ± 0.089	0.713 ± 0.092	*t* = 2.22	.028*
Lumbar spine T-score	−2.19 ± 0.78	−1.86 ± 0.72	*t* = 2.65	.009*
Total hip T-score	−1.84 ± 0.69	−1.55 ± 0.63	*t* = 2.50	.014*
Prevalence of vertebral fractures, n (%)	19 (30.6)	13 (14.8)	χ^2^ = 5.59	.018*
Multiple vertebral fractures, n (%)	8 (12.9)	4 (4.5)	χ^2^ = 4.02	.045*

T-score assessed by dual-energy X-ray absorptiometry.

BMD = bone mineral density, ILD = interstitial lung disease, RA = rheumatoid arthritis.

* *P* < .05 indicates statistical significance.

### 3.3. ILD severity and BMD

Among RA-ILD patients, BMD progressively declined with increasing ILD severity (mild → moderate → severe; Table 3). Lumbar spine BMD in patients with mild, moderate, and severe ILD was 0.892 ± 0.110, 0.866 ± 0.106, and 0.801 ± 0.095 g/cm^2^, respectively, with statistically significant differences among the 3 groups (F = 4.62, *P* = .012) and a significant linear trend (*P* for trend = .006). Lumbar spine T-scores also showed a decreasing trend (−1.95 ± 0.74 vs −2.22 ± 0.69 vs −2.51 ± 0.70, *F* = 4.21, *P* = .018, *P* for trend = .008). Although total hip BMD and T-scores did not differ significantly among groups (*P* = .077 and 0.065, respectively), both exhibited a decreasing trend with worsening ILD severity (*P* for trend = .031 and .043). Femoral neck BMD differed significantly across ILD severity (*F* = 3.14, *P* = .049, *P* for trend = .024), indicating that bone loss in weight-bearing sites was also notable. Pulmonary function parameters were positively correlated with BMD (Table 4). Forced vital capacity (%) was significantly correlated with lumbar spine BMD (*r* = 0.28, *P* = .032), and diffusing capacity for carbon monoxide (%) was significantly correlated with total hip BMD (*r* = 0.30, *P* = .025), suggesting that greater impairment in lung diffusion function was associated with more pronounced bone loss. Overall, ILD severity was associated with progressive bone loss, particularly in the lumbar spine, indicating a dose-response relationship between ILD progression and skeletal health deterioration.

**Table 3:** Bone mineral density among RA patients with different ILD severity levels.

Parameter	Mild ILD (n = 27)	Moderate ILD (n = 24)	Severe ILD (n = 11)	*F*/χ^2^	*P* value	*P* for trend
Lumbar spine BMD (g/cm^2^)	0.892 ± 0.110	0.866 ± 0.106	0.801 ± 0.095	4.62	.012*	.006*
Total hip BMD (g/cm^2^)	0.802 ± 0.099	0.783 ± 0.102	0.748 ± 0.088	2.67	.077	.031*
Femoral neck BMD (g/cm^2^)	0.701 ± 0.087	0.687 ± 0.091	0.654 ± 0.083	3.14	.049*	.024*
Lumbar spine T-score	−1.95 ± 0.74	−2.22 ± 0.69	−2.51 ± 0.70	4.21	.018*	.008*
Total hip T-score	−1.63 ± 0.65	−1.82 ± 0.67	−2.03 ± 0.60	2.84	.065	.043*

Data are presented as mean ± SD.

BMD = bone mineral density, ILD = interstitial lung disease, RA = rheumatoid arthritis, SD = standard deviation.

* *P* < .05 was considered statistically significant.

**Table 4:** Correlation between pulmonary function and bone mineral density in RA-ILD patients.

Parameter	Lumbar spine BMD	Total hip BMD	Femoral neck BMD
FVC%	*r* = 0.28, *P* = .032*	*r* = 0.21, *P* = .097	*r* = 0.19, *P* = .123
DLCO%	*r* = 0.23, *P* = .061	*r* = 0.30, *P* = .025*	*r* = 0.26, *P* = .038*

BMD = bone mineral density, DLCO% = diffusing capacity of the lung for carbon monoxide (% predicted), FVC% = forced vital capacity (% predicted), ILD = interstitial lung disease, RA = rheumatoid arthritis.

* *P* < .05 was considered statistically significant.

### 3.4. Multivariable logistic regression analysis of ILD severity and OVF risk

To assess the independent effect of ILD severity on the risk of OVFs in RA patients, a multivariable logistic regression model was constructed (Table 5). ILD severity was treated as an ordinal variable (mild = 1, moderate = 2, severe = 3), and potential confounders, including age, sex, BMI, cumulative glucocorticoid dose, DAS28-CRP, and use of bone-protective therapy, were adjusted for. The results demonstrated that ILD severity was an independent risk factor for OVF: for each 1-level increase in ILD severity, the odds of vertebral fracture increased approximately 2.51-fold (OR = 2.51, 95% CI: 1.21–5.22, *P* = .014). Additionally, age (per 10-year increase, OR = 1.38, 95% CI: 1.04–1.83, *P* = .027) and cumulative glucocorticoid dose (per 1 g increase, OR = 1.22, 95% CI: 1.05–1.42, *P* = .009) were also independent risk factors. Sex, BMI, DAS28-CRP, and bone-protective therapy were not significantly associated with OVF risk in this model (all *P* > .05). Overall, ILD severity independently predicted OVF in RA patients, highlighting the need for heightened clinical attention to bone health and fracture risk in patients with progressive ILD.

**Table 5:** Multivariate logistic regression analysis of independent risk factors for osteoporotic vertebral fractures in RA patients.

Variable	OR	95% CI	*P* value
ILD severity (mild → severe)	2.51	1.21–5.22	.014*
Age (per 10-yr increase)	1.38	1.04–1.83	.027*
Sex (female vs male)	1.21	0.56–2.61	.626
BMI (kg/m^2^)	0.92	0.80–1.05	.204
Cumulative glucocorticoid dose (g)	1.22	1.05–1.42	.009*
DAS28-CRP	1.14	0.87–1.49	.345
Bone-protective therapy (yes vs no)	0.83	0.37–1.87	.657

BMI = body mass index, CI = confidence interval, DAS28-CRP = Disease Activity Score 28-CRP, ILD = interstitial lung disease, OR = odds ratio, RA = rheumatoid arthritis.

* *P* < .05 indicates statistical significance.

### 3.5. Association between ILD severity and OVF grading

Among the 150 RA patients included in this study, 62 patients had RA-ILD, classified by HRCT imaging into mild (n = 27), moderate (n = 24), and severe (n = 11) groups. Vertebral fracture severity was assessed using the Genant semiquantitative method, and OVFs were categorized as mild (grade 1), moderate (grade 2), or severe (grade 3). The results demonstrated a clear upward trend in both the incidence and severity of vertebral fractures with increasing ILD severity (Table 6). The OVF incidence in patients with mild ILD was 14.8%, all of which were mild fractures, with no moderate or severe fractures observed. In moderate ILD, the OVF incidence was 29.2%, with 57.1% mild fractures and 42.9% moderate fractures. In severe ILD, OVF incidence reached 54.5%, with 18.2% mild, 45.5% moderate, and 36.3% severe fractures. Trend analysis indicated that both OVF incidence and fracture severity significantly increased with ILD progression (*P* for trend = .008). Further analysis revealed a strong correlation between ILD grade and OVF severity, indicating that patients with more advanced ILD not only have a higher risk of vertebral fractures but also tend to experience more severe fractures.

**Table 6:** Vertebral fracture incidence and severity according to ILD severity in RA patients.

ILD severity	OVF incidence n (%)	Grade 1 n (%)	Grade 2 n (%)	Grade 3 n (%)	*P* for trend
Mild (n = 27)	4 (14.8%)	4 (100%)	0 (0%)	0 (0%)	.008*
Moderate (n = 24)	7 (29.2%)	4 (57.1%)	3 (42.9%)	0 (0%)	
Severe (n = 11)	6 (54.5%)	1 (18.2%)	3 (45.5%)	2 (36.3%)	

*P* for trend was calculated using the Cochran–Armitage trend test.

Grade 1 = mild fracture, Grade 2 = moderate fracture, Grade 3 = severe fracture, ILD = interstitial lung disease, OVF = osteoporotic vertebral fracture, RA = rheumatoid arthritis.

* *P* < .05 indicates statistical significance.

## 4. Discussion

In this study of 150 RA patients, we systematically analyzed BMD and the occurrence of OVFs in RA patients with ILD, further exploring the association between ILD severity and skeletal health. The results demonstrated that RA-ILD patients exhibited significantly lower BMD and higher OVF incidence compared with RA patients without ILD. Notably, both BMD loss and fracture risk increased progressively with ILD severity, displaying a clear dose-response relationship. Multivariable logistic regression analysis confirmed ILD as an independent risk factor for OVFs in RA patients, highlighting the need for heightened attention to bone health in this population.

Several mechanisms may underlie the observed bone loss in RA-ILD patients. First, systemic inflammation is more pronounced in RA-ILD patients, as evidenced by elevated CRP and ESR levels in this study. Chronic inflammation can promote osteoclast activity and inhibit osteoblast function via the RANKL/OPG signaling pathway, accelerating bone loss.^[12,13]^ Second, RA-ILD patients had higher cumulative glucocorticoid exposure, which can directly suppress osteoblast activity, increase osteoclast-mediated bone resorption, and disrupt calcium and phosphate metabolism, further increasing OP risk.^[14,15]^ Third, ILD-related pulmonary impairment and activity limitation may indirectly contribute to bone loss. Our findings showed that forced vital capacity (%) correlated positively with lumbar BMD, and diffusing capacity for carbon monoxide (%) correlated positively with total hip BMD, suggesting that reduced pulmonary diffusing capacity may lead to decreased mechanical loading and oxygen delivery, thereby promoting bone loss, which is consistent with previous reports.^[16,17]^

A key finding of this study is the dose-response relationship between ILD severity and both BMD reduction and fracture risk. In RA-ILD patients, lumbar spine and femoral neck BMD decreased progressively from mild to severe ILD, and the OVF incidence in the severe group reached 54.5%, with the highest proportion of moderate-to-severe fractures. Multivariable logistic regression showed that each 1-grade increase in ILD severity was associated with a 2.51-fold increase in OVF risk. These results indicate that ILD progression not only exacerbates bone loss but also increases fracture severity. This aligns with prior studies linking chronic inflammation in RA to fracture risk^[18,19]^ and provides novel evidence supporting ILD severity as an independent risk factor for vertebral fractures.

From a clinical perspective, our findings suggest that RA-ILD patients, particularly those with moderate-to-severe ILD, should undergo early BMD assessment and fracture risk screening. High-risk patients may benefit from individualized interventions, including early anti-osteoporotic therapy (e.g., calcium, vitamin D, bisphosphonates, or denosumab), disease activity control, and optimized glucocorticoid regimens.^[20]^ Additionally, maintaining pulmonary function and engaging in rehabilitation may indirectly improve bone mass and reduce fracture risk.

This study has several limitations. First, it is a single-center retrospective study with a relatively small sample size, particularly in the severe ILD subgroup, which may affect statistical precision. Second, some vertebral fractures may have been missed by DXA or imaging, leading to potential underestimation. Third, bone microarchitecture and strength parameters were not evaluated; future studies incorporating HR-pQCT or bone morphometric analyses could provide further validation. Nevertheless, this study leveraged clinical, imaging, and laboratory data to systematically analyze the relationship between ILD severity, BMD, and OVF risk, providing evidence to guide bone health management in RA-ILD patients.

In conclusion, RA patients with ILD exhibit significantly reduced BMD, and both the incidence and severity of OVFs increase progressively with ILD severity. ILD is an independent risk factor for vertebral fractures in RA, underscoring the importance of enhanced bone health assessment and targeted management strategies in this population.

## Author contributions

**Conceptualization:** Na Li, Jiaxin Li.

**Funding acquisition:** Na Li, Jiaxin Li.

**Data curation:** Na Li, Jiaxin Li.

**Formal analysis:** Na Li, Jiaxin Li.

**Investigation:** Na Li, Jiaxin Li.

**Writing – original draft:** Na Li, Jiaxin Li.

**Writing – review & editing:** Na Li, Jiaxin Li.
